# Activity Patterns of Free-Ranging Koalas (*Phascolarctos cinereus*) Revealed by Accelerometry

**DOI:** 10.1371/journal.pone.0080366

**Published:** 2013-11-04

**Authors:** Michelle A. Ryan, Desley A. Whisson, Greg J. Holland, John P. Y. Arnould

**Affiliations:** Centre for Integrative Ecology, School of Life and Environmental Sciences, Deakin University, Burwood, Victoria, Australia; University of Western Ontario, Canada

## Abstract

An understanding of koala activity patterns is important for measuring the behavioral response of this species to environmental change, but to date has been limited by the logistical challenges of traditional field methodologies. We addressed this knowledge gap by using tri-axial accelerometer data loggers attached to VHF radio collars to examine activity patterns of adult male and female koalas in a high-density population at Cape Otway, Victoria, Australia. Data were obtained from 27 adult koalas over two 7-d periods during the breeding season: 12 in the early-breeding season in November 2010, and 15 in the late-breeding season in January 2011. Multiple 15 minute observation blocks on each animal were used for validation of activity patterns determined from the accelerometer data loggers. Accelerometry was effective in distinguishing between inactive (sleeping, resting) and active (grooming, feeding and moving) behaviors. Koalas were more active during the early-breeding season with a higher index of movement (overall dynamic body acceleration [ODBA]) for both males and females. Koalas showed a distinct temporal pattern of behavior, with most activity occurring from mid-afternoon to early morning. Accelerometry has potential for examining fine-scale behavior of a wide range of arboreal and terrestrial species.

## Introduction

An understanding of a species’ activity patterns is important for its conservation. Activity may be adapted according to biotic factors such as resource availability, competition and predation risk, or abiotic factors such as photoperiod and ambient temperature (e.g., [1-3]). For example, more time may be spent in foraging where there is low availability of food resources [4], or activity may be restricted to the coolest times of the day where climates are warmer (e.g., [5]). Any increase in activity with environmental change may incur higher energetic costs and, thus, have an influence on population ecology (e.g., reduced breeding effort, increased mortality rates). Despite its importance, activity patterns are poorly understood for many species due to the limitations of traditional field methodologies.

As a specialist folivore feeding exclusively on *Eucalyptus* spp., the koala (*Phascolarctos cinereus*) is particularly susceptible to changes in its environment [6]. The low nutritive value and high fibre content of *Eucalyptus* foliage, together with the cost of detoxifying secondary plant compounds, interact to limit available energy for the species [7-9]. Consequently, koalas, like many other arboreal herbivores, remain inactive for long periods to conserve energy and aid digestion [10-12]. It is therefore important to understand how koalas apportion time to different behaviors such as feeding and resting, and the factors influencing their activity patterns. 

An understanding of koala activity patterns remains a major knowledge gap, with most research to date being undertaken on captive animals [13-18], or in field studies involving limited observations of a few individuals over short time periods (e.g., [11,12,19]). These studies indicate that there is high inter-individual variation in activity due to age, gender and physiological state. In free-ranging populations, factors such as weather, food resources and population density also may influence the activity of individuals, leading to significant variance and lack of analytical power when sample sizes are small. Continuous observation of large numbers of individuals for extended periods, however, is logistically difficult and time-consuming. 

 Accelerometry is gaining popularity as a tool for indirectly measuring activity in free-ranging animals (e.g., [20-23]). Accelerometer data loggers continuously measure acceleration on up to three axes for an extended period of time. Traditionally used in human studies (e.g., [24-26]), accelerometer data loggers are now being developed for attachment to animals. When attached along an animal’s trunk, multi-axis accelerometer data loggers accurately record its overall body movement [20,23]. For some species, this has provided detailed information on the timing and duration of different activities (e.g., red-footed booby, *Sula sula* [27]). Furthermore, data from the axes may be combined to derive an estimate of overall dynamic body acceleration (ODBA [23]). Studies suggest that ODBA is highly correlated with energy expenditure in many species [21]. 

Use of accelerometer data loggers may therefore provide a means of indirectly recording activity in koalas. The aims of this study were to: (1) develop a method for examining activity patterns of koalas, using accelerometry; and (2) apply this method to examining activity of male and female koalas in a high density population of koalas during the breeding season. 

## Methods

### Ethics statement

This study was approved by the Deakin University Animal Ethics Committee (A31-2010) and conducted under permit (10005379) by the Victorian Department of Sustainability and Environment. Permission to access private property was obtained from landholders C. and P. Marriner. 

### Study area, animal handling and instrumentation

**Figure 1:**
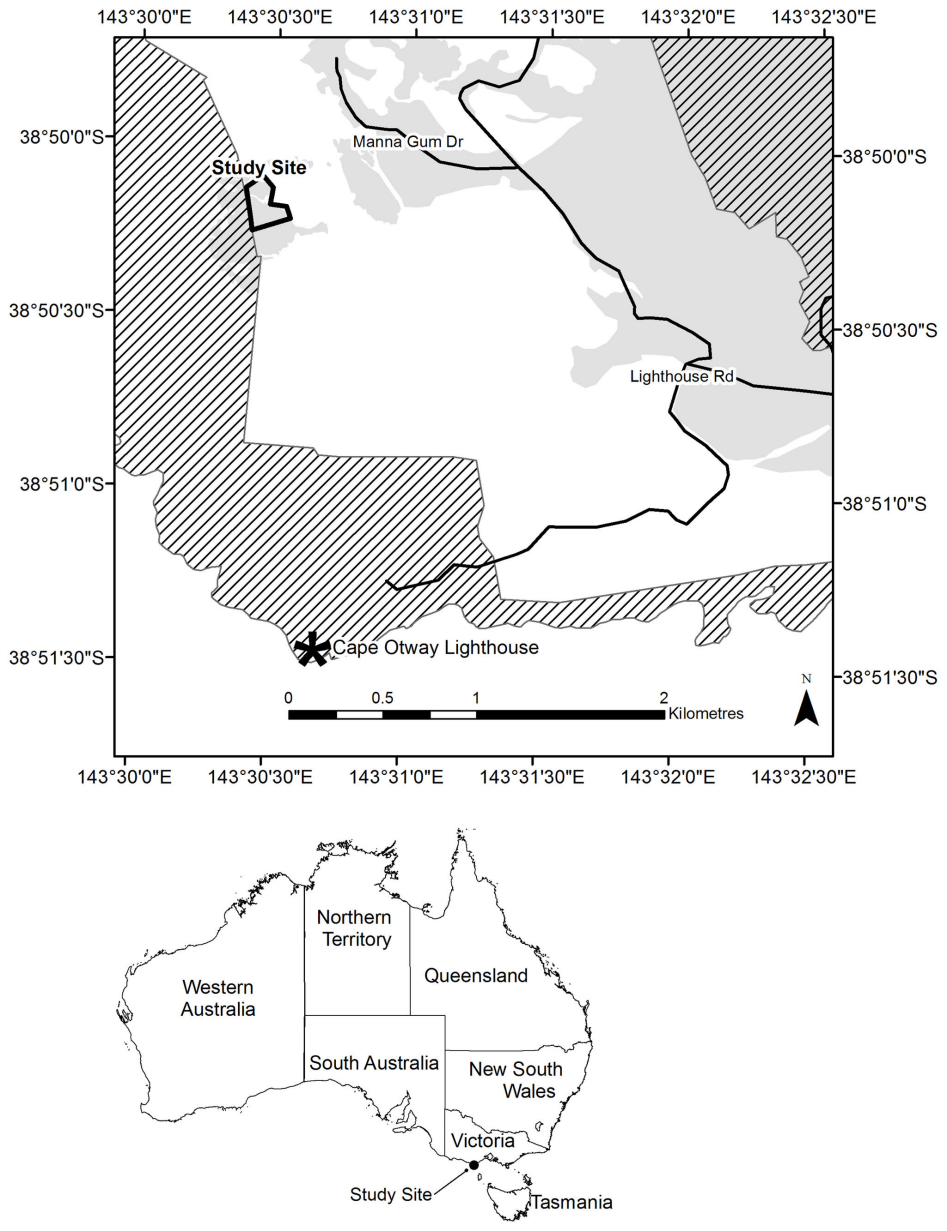
Location of the study site at Cape Otway, Victoria. Great Otway National Park is shown in hatching and forest/woodland areas in grey.

The study was undertaken in a 7-ha manna gum (*Eucalyptus viminalis*) woodland on private property at Cape Otway (38°50’06”S, 143°30’25”E), Victoria, Australia (Figure 1). Average annual rainfall is 898 mm and temperatures range from a mean monthly minimum of 7.5°C in July to a mean monthly maximum of 21.6°C in February (Bureau of Meteorology website, Station ID 090015. Available: http://www.bom.gov.au. Accessed 2013 Oct 4). 

 Sampling was undertaken in two periods during the koala breeding season: 1-13 November 2010 (early-breeding) and 21 - 31 January 2011 (late-breeding). Koala density at the site was assessed on one afternoon (13:00h - 17:00h) during each sampling period. Two observers systematically searched all trees in a 2-ha area within the site and recorded the number of koalas sighted. The gender of each koala, and the presence of dependent young with adult females, was recorded during these surveys. 

Twelve koalas were fitted with accelerometer data loggers in the early sampling period and a different set of 15 koalas used in the late sampling period. Koalas were captured during the day using a standard noose and flag technique (see 28). Only sexually mature koalas without dependent young were used (>6 kg [29]). Each individual was marked with a uniquely numbered ear-tag and its weight recorded. A VHF radio collar (Sirtrack, Havelock North, NZ; 100g) with tri-axial accelerometer data logger (Gulf Coast Data Concepts LLC, Waveland, MS 39576; model X6-1A; 55g; 41x10.1x10.4mm) was fitted to each koala. The accelerometer data logger was programmed to record at 20 Hz with 12-bit resolution and encased in rubber for waterproofing. It was attached with fabric tape to the radio collar of each koala. Each accelerometer was fitted to the collar so that the x-axis was along the spine of the animal. Koalas were processed at their capture points and then released. Each koala was located at least twice (morning and evening) each day, and its locations recorded with a handheld global positioning system (GPS) (Garmin, Kansas, U.S.A.). All individuals were recaptured 7 d after initial capture and their collars removed.

 To determine the effect of branches moving in the wind on the accelerometer data, two accelerometer data loggers were placed in a randomly-selected tree within the site during the late-breeding sampling period. One accelerometer data logger was attached to the tree trunk (3 m above the ground) and the other at the end of a branch (5 m above the ground and 4 m from the main trunk). These locations were chosen as representative of koala resting locations.

 To assess the effectiveness of accelerometer data loggers in detecting particular behaviors, visual observations of a random sample of 16 study animals (both males and females and from both sampling periods) were conducted. These were conducted in 15 min blocks up to seven times (Mean = 4) during the day, for a total of 63 h of observation. Koalas were observed from a distance of approximately 15 m, at which the behavior of the koala was not influenced by the observer. Behaviors were recorded in 1-s intervals and coded according to an ethogram (adapted from [30]; see Table 1). 

**Table 1:** Koala ethogram (adapted from [30]).

**Behavior**	Description
**Sleeping**	Head tucked down and no movement
**Rest-alert**	Head up and eyes open or closed. Sedentary but may include head movements, stretching or repositioning.
**Feeding**	Sniffing leaves, reaching for foliage, chewing.
**Moving**	Movement up and down trees, along branches or along the ground.
**Grooming**	Scratching of body.
**Vocalising**	Bellows, screams.

 To determine the effect of temperature on activity patterns of koalas, hourly temperature data for each sampling period were obtained from the BOM weather station (Station ID 090015) located approximately 5 km from the study site.

### Data Analysis

Accelerometer data were processed to provide an index of movement for each second, known as Overall Dynamic Body Acceleration (ODBA g.s^-1^ [23]). The observations of koala behavior were time-matched with the accelerometer data. The mean ODBA index was calculated for each behavior (*sleeping*, *rest-alert*, *feeding*, *moving*, *grooming*) displayed by each koala (see Table 2). ODBA indices for these behaviors were then compared in a mixed model, with koala specified as a random term. Based on this analysis, seconds were classified as either *inactive* or *active* and periods of activity for each koala defined (see Results: ‘Developing an Activity Index’). 

**Table 2:** Mean ODBA (gravitational force per second [g.s^-1^]) for behaviors.

	Behavior	ODBA·s^-1^		
		Mean	SE	N
Inactive behaviors (ODBA < 1)	Sleeping	0.275	0.06	14
	Rest alert	0.324	0.04	16
Active behaviors (ODBA > 1)	Feeding	2.125	0.18	11
	Moving	4.053	0.64	5
	Grooming	2.239	0.19	7

N is the number of koalas observed exhibiting the behavior. The limited number of observations of koalas vocalising precluded determining ODBA for that behavior.

 Generalized additive mixed models (GAMM [31,32]) were used to investigate the relationship between time spent in active behaviors and hour of day, animal gender, and survey period. GAMMs employ smoothing functions that allow non-linear relationships to be modelled. Inspection of raw data suggested that koala activity patterns may vary through time in a non-linear manner. Mixed models allow the inclusion of random effects to account for correlation structure in the data [32]. Such an approach was required here due to the repeat sampling of individual animals.

 For each animal included in the study, time spent in active behaviors (minutes) was calculated for each hour (n = 24) of each day (n = 7). Because of the high frequency of zeros (i.e., no activity in an hour) for individuals, hourly averages (mean minutes active per hour) were calculated, resulting in a dependent variable that consisted of 24 values for each animal studied. This was modelled using a Poisson error distribution. Hour of day was included as a non-linear smoothed term, with the degree of smoothing determined internally during the fitting process [31]. Both animal gender and survey period were included as categorical linear terms. Hourly temperature was collinear with time of day so was excluded as a term. Interactions between hour of day and the two categorical terms were investigated by fitting separate smoothed terms for hour of day for each level of gender and survey period (via the use of variable coefficient GAMMs [32]). An identifier for individual koalas was included as a random effect. The fit of a model with and without a temporal correlation structure was also assessed to investigate the possibility that, for any given individual, values closer in time may be more strongly correlated than those farther apart [32]. Model fit was assessed by calculating the percentage of deviance explained using modified script (from [33]). These analyses were performed using the ‘mgcv’ package ver. 1.7-18 [31] in R ver. 2.15.1 [34].

 From daily location data collected for each koala, the standard distance (one standard deviation from the mean centre) of daily locations, mean daily distance moved, and numbers of trees used during sampling periods were analysed using 2-way ANOVA to determine differences in ranging behavior between genders, sampling periods, and the interaction of these effects. Differences in ODBA between genders and sampling periods were also examined using 2-way ANOVA. Pearson’s correlation coefficient was used to examine the relationship between temperature and number of minutes per hour active. These analyses were conducted using SPSS 21.0. Data are presented as Mean ± SE.

## Results

 There were 16 and 12.5 koalas per hectare in the study area in the early- and late-breeding sampling periods, respectively. In the early-breeding period, 67% of females had back-young, whereas in the late-breeding period, only 25% had back-young present. 

### Developing an Activity Index

Gentle to moderate breezes (average = 20km·h^-1^) were observed during both sampling periods. Wind movement of trees had little effect on ODBA (g.s^-1^) in January with mean values of 0.038 g·s^-1^ and 0.245 g·s^-1^ for the tree trunk and branch accelerometers, respectively. Furthermore, mean ODBA (g·s^-1^) for sleeping koalas was similar between sampling periods (t_25_ = 1.67, *P* = 0.34), suggesting that wind movement of trees on accelerometer data for koalas was also negligible in November.

**Figure 2:**
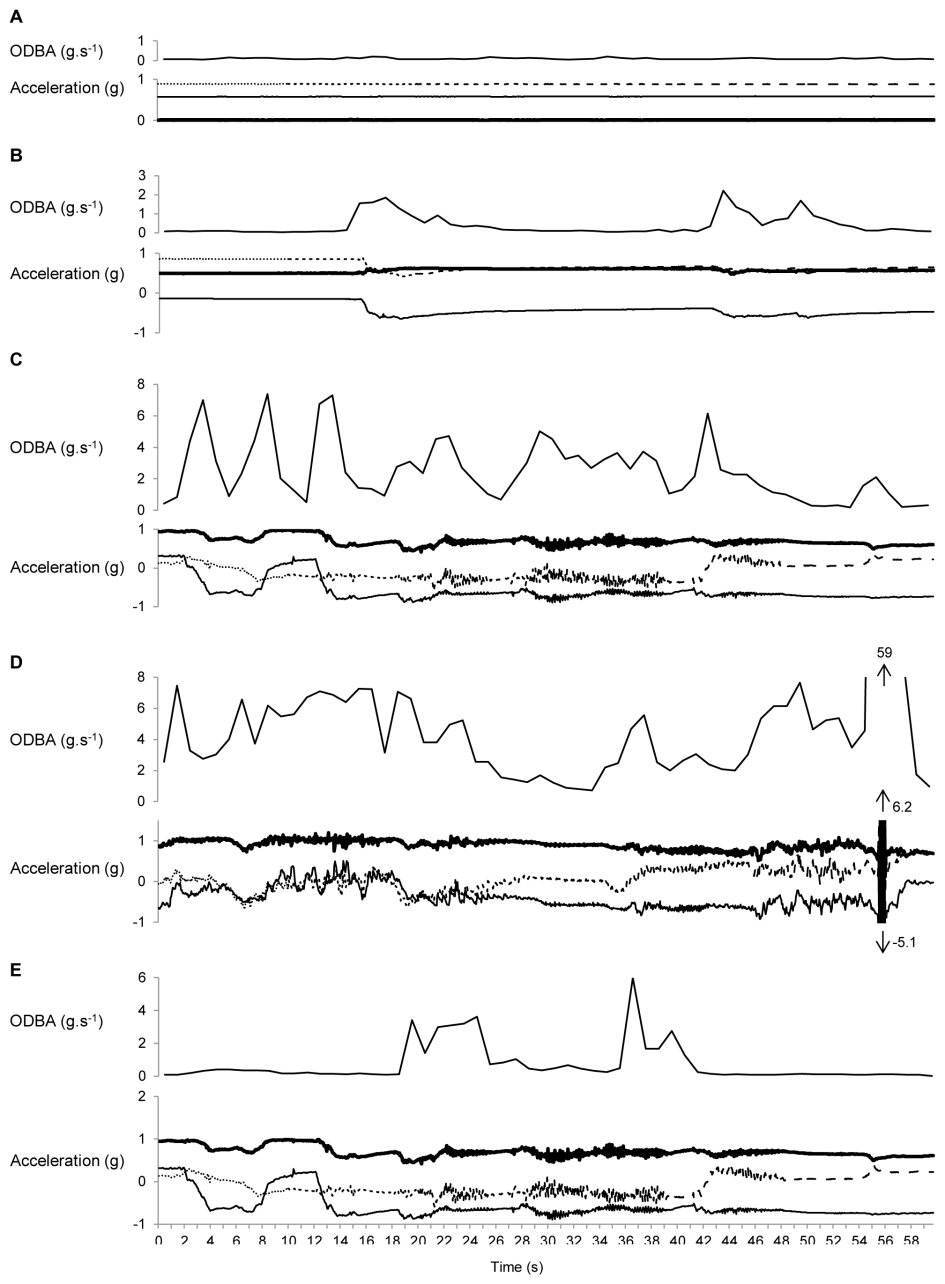
Accelerometer traces and Overall Dynamic Body Acceleration (ODBA) for A. Sleeping, B. Rest Alert, C. Feeding, D. Moving, E. Grooming. Traces are acceleration (gravitational force·second^-1/20^) on each of three axes for 60 seconds of each behavior. Accelerometer axes are: x-axis = thin solid line, y-axis = dotted line, z-axis = thick solid line.

Accelerometer data reflected koala posture and movement associated with different behaviors (Figure 2). When *sleeping*, koalas remained in a tucked, motionless position such that acceleration was negligible on all axes and ODBA (g·s^-1^) was near zero. *Rest-alert* behavior was characterised by occasional movements of up to 10 s duration, with relatively low level acceleration on any axis and low ODBA (g.s^-1^). *Feeding* and *moving* were usually observed in combination as koalas moved within canopies to locate preferred foliage. While *feeding*, koalas alternated between periods of activity (31-107 s) and inactivity (up to 48 s). Both *feeding* and *moving* were associated with irregular, high-amplitude changes in g-force on all axes, and irregular ODBA values. High acceleration was associated with rapid movement, e.g., jumping between branches (Figure 2, *moving*). *Grooming* (usually scratching) occurred in short bursts of 5-10 s during rest-alert periods, and was characterised by an irregular pattern of acceleration on all axes and, thus, an irregular pattern of ODBA (g.s^-1^). There were insufficient observations of *vocalisations* to identify a pattern for this behavior. 

ODBA (g·s^-1^) varied between behaviors (F_4,45.75_ = 55.62, *P* < 0.001). Active behaviors (*feeding*, *moving* and *grooming*) were associated with a mean ODBA greater than 2.1 (g·s^-1^), and inactive behaviors (*sleeping* and *rest alert*) were associated with a mean ODBA less than 0.5 g·s^-1^ (Table 2). Periods of activity were characterised by periods of high movement (and thus high ODBA values) interspersed with short periods of inactivity. Consequently, to define activity bouts, it was necessary to apply a smoothing filter. Firstly, ODBA (g·s^-1^) was square-root transformed to reduce the effect of rapid momentary acceleration (i.e., high ODBA values), and then a 5-minute running mean was applied to remove short periods of inactivity within activity bouts. Based on observed mean values of ODBA (g·s^-1^) for active behaviors (*feeding*, *moving*, *grooming*; Table 2), smoothed ODBA values > 1.0 g·s^-1^ were classified as active behaviors, and values <1.0 g·s^-1^ were classified as inactive behaviors (*sleeping* and *rest-alert*). The effectiveness of this approach was tested by comparing the timing of predicted behaviors with observed behaviors. Almost all (97%) *feeding*, *moving*, and *grooming* seconds were correctly identified as occurring during active periods, and 99% of *sleeping* and *rest-alert* seconds were correctly identified as occurring during inactive periods.

###  Spatial, seasonal and diel variation in activity

The standard distance of each koala’s locations was greater in the early-breeding period (28.9 ± 4.5 m) than in the late-breeding period (19.6 ± 3.3 m), but did not vary between sexes (Table 3). The mean daily distance moved was also greater in the early-breeding period than the late-breeding period, with males moving greater distances than females in both periods (Table 3). The number of trees used did not vary between sample periods but male koalas used more trees (7.1 ± 0.7 trees) than females (4.8 ± 0.5 trees). 

**Table 3:** Activity and ranging behavior of male and female koalas during the early- and late-breeding seasons.

Characteristic	Early breeding		Late breeding		ANOVA results
	Male	Female	Male	Female	
Mean median length of activity bouts (minutes)	16.2 + 1.60	17.3 + 4.90	16.5 + 0.99	20.8 + 3.92	Season: *F*_1,23_ = 0.48, *P* = 0.50
					Sex: *F*_1,23_ = 0.37, *P* = 0.55
					Season*Sex: *F*_1,23_ = 0.97, *P* = 0.34
Time per day spent in activity (minutes)	360 + 15.8	351 + 25.5	330 + 23.0	314 + 13.4	Season: *F*_1,23_ = 3.11, *P* = 0.09
					Sex: *F*_1,23_ = 0.46, *P* = 0.50
					Season*Sex: *F*_1,23_ = 0.03, *P* = 0.85
Mean total daily ODBA (g x 10^4^)	6.24 + 0.22	5.90 + 0.50	5.47 + 0.26	5.34 + 0.23	**Season: *F*_1,23_ = 5.11, *P* = 0.03^a^**
					Sex: *F*_1,23_ = 0.63, *P* = 0.44
					Season*Sex: *F*_1,23_ = 0.13, *P* = 0.73
Standard distance (m)	31.59 + 5.85	25.45 + 6.69	18.14 + 4.04	17.14 + 4.46	**Season: *F*_1,23_ = 4.65, *P* = 0.04^a^**
					Sex: *F*_1,23_ = 2.01, *P* = 0.17
					Season*Sex: *F*_1,23_ = 1.21, *P* = 0.28
Daily distance moved (m)	58.81 + 13.06	13.56 + 3.77	20.57 + 7.02	11.78 + 3.93	**Season: *F*_1,23_ = 5.62, *P* = 0.03^a^**
					**Sex: *F*_1,23_ = 9.76, *P* = 0.005^a^**
					Season*Sex: *F*_1,23_ = 3.50, *P* = 0.07
Trees used per week	7.7 + 0.9	4.4 + 0.6	6.3 + 0.9	5.0 + 0.7	Season: *F*_1,23_ = 0.22, *P* = 0.65
					**Sex: *F*_1,23_ = 7.70, *P* = 0.01^a^**
					Season*Sex: *F*_1,23_ = 1.40, *P* = 0.25

^a^Significant at *P* = 0.05

The duration of activity bouts did not vary between sampling periods, sexes or with the interaction of these effects (Table 3). Activity bouts ranged in duration from 1 minute to 6.4 hours, with a mean median length of 17.5 minutes. Koalas tended to spend more time per day in active behaviors in the early-breeding season (5.9 h·day^-1^) than in the late-breeding season (5.4 h·day^-1^; Table 3). Mean total daily ODBA (g·day^-1^) was higher in the early-breeding season than in the late-breeding season (Table 3). 

 In the analysis of temporal activity, inclusion of a temporal correlation structure (auto-regressive correlation; AR-1) improved the fit of the GAMM (AIC with correlation structure =1148.3; AIC without correlation structure =1232.8). Results are, therefore, based on the model including this correlation structure. There was no evidence of an interaction between hour of day and the two categorical variables (gender and season). To aid interpretation, results are presented for the model in which no interactions were included.

**Figure 3:**
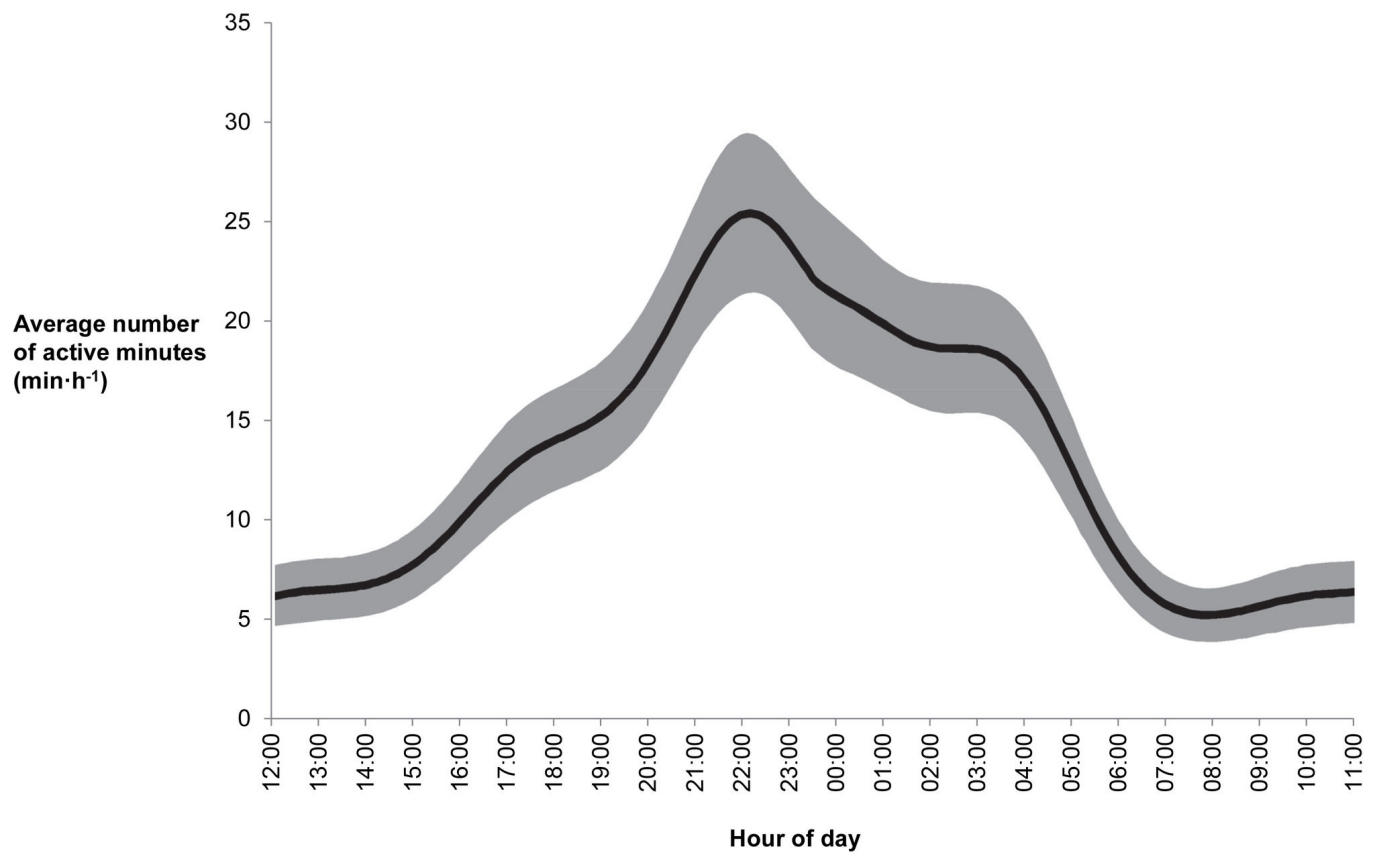
The average number of minutes in which koalas were active as a function of hour of day. The solid line represents predicted values from a generalized additive mixed model (GAMM), while the shaded region depicts the 95% confidence interval around predictions.

 The generalized additive mixed model explained 42% of variance in the data. Hour of day was the only variable found to be an important influence on the average number of minutes per hour spent active by koalas (Table 4). Koalas were most active between 20:00 - 04:00 h. A decline in activity was observed between 04:00 - 07:00 h. Koalas were relatively inactive between 07:00 - 1400 h, after which activity increased to the evening peak (Figure 3). The mean number of minutes per hour spent in active behaviors was negatively correlated with mean hourly temperature in both sampling periods (November: r_p_ = -0.65, *P* = 0.001; January: r_p_ = -0.43, *P* = 0.03).

**Table 4:** Results from a generalized additive mixed model (GAMM) describing the relationship between the average number of minutes in which koalas are active and hour of day, animal gender, and survey period (early- and late-breeding season).

Explanatory variable	e.d.f.^a^	*F*	*P*-value
*s*(Hour of day)^b^	7.87	27.33	<0.001
Gender: male^c^	n.a.^e^	0.78^f^	0.433
Survey period: late breeding^d^	n.a.^e^	-1.68^f^	0.094

^a^Estimated degrees of freedom for non-linear smoothed terms

^b^Smoothed term fitted for hour of day

^c^The reference category for the categorical variable gender was female

^d^The reference category for the categorical variable survey period was early breeding season

^e^Estimated degrees of freedom not applicable for parametric terms (i.e., variables not fitted with smoothing terms

^f^T-values are used to test the significance of parametric terms

## Discussion

 Our study has demonstrated that accelerometry is an effective tool for examining activity patterns of an arboreal species. Accelerometer data loggers are small and easily attached to radiocollars and, thus, do not interfere with natural activity of individuals. They provide fine-scale, continuous, multi-day data on animal activity such that large numbers of individuals can be monitored simultaneously. To date, small sample sizes have been typical in studies of animal behavior and, therefore, may not have accounted for high individual variation in behavior [35]. This has been the case with studies of koala activity. For example, one study [11] reported time budgets based on direct observations of four female koalas for 24 hours; and another [19,20] used acoustic devices to monitor activity of five female and six male koalas over 24-h periods. In our study, accelerometers allowed us to examine activity of 27 koalas for 7-d periods, equivalent to a total of 4536 observation hours. We identified a clear diel cycle of activity with most activity occurring during the coolest hours of the day. This pattern did not vary between genders or phase of the breeding season, but overall activity tended to be higher during the early-breeding season.

 Accelerometry is gaining popularity as a tool for determining the timing and frequency of specific behaviors in many wildlife species. For example, a pattern in accelerometer traces was identified for walking in cormorants (*Phalacrocorax carbo*) [23]; and a unique pattern of head movement was associated with prey encounter in penguins (*Pygoscelis* spp.) [36]. In our study with koalas, we were not able to discern specific accelerometer traces associated with any particular activity that would assist in their identification. Direct observation of koalas suggests that this was due to their relatively erratic movements during all active behaviors. While feeding, koalas alternate between short bursts of high movement (e.g., reaching for leaves, moving to new locations in the tree), and periods of relative inactivity (chewing, observing). Feeding, moving to new roosting points within a tree, and grooming had similar accelerometer traces. Although we are unable to validate this, it may be possible to identify male ‘bellow’ vocalisations as they are associated with a unique, repetitive head movement. Vocalisations play an important role in mate selection and male territoriality (e.g., [37,38]) and, therefore, an ability to determine their duration, timing and frequency would be valuable in understanding aspects of breeding behavior.

Whereas we could not detect specific behaviors from the accelerometer data, we were able to distinguish between inactive and active periods for each individual, based on an index of activity (ODBA) derived from the three accelerometer axes. While sleeping or resting, the index of activity was low (ODBA < 1 g·s^-1^) whereas it was up to ten times higher when koalas were feeding, moving or grooming (active behaviors). To define activity bouts, a smoothing filter was applied so that short periods of inactivity (up to 48 s, see Figure 2) within activity bouts were correctly assigned. This allowed us to determine the total amount of time koalas were active per day, and to characterise activity patterns of the study animals, for comparison of the timing and duration of activity bouts between genders and season.

 Koalas were active for an average of 5 - 6 h per day which is at the upper end of the range previously reported (3.6 to 5 hours per day [11,14,19,39]). Previous studies that have been based on captive animals, or on limited observation of free-ranging individuals, may have underestimated activity. Captive koalas are held in small enclosures, do not need to search for food, and are housed to prevent aggressive interactions [40]. In studies of free-ranging koalas that exhibit high daily and inter-individual variation in activity, activity estimates based on limited observations may not be representative. Indeed, the high individual variation in total daily activity observed in both sampling periods in our study (4.5 - 7 h·d^-1^) highlights the need for large sample sizes in studies of koala activity.

 A number of factors may also have contributed to the high levels of koala activity in our study. The study was conducted during the breeding season in an area of extremely high koala population density (>12 koalas per hectare), reflecting abundant availability of high quality food resources (i.e., manna gum [8]). Although koalas moved only short distances (<60 m) within a small area during the 7-d study periods (standard distance of locations <32 m), interactions between individuals were potentially frequent due to the high population density and heightened breeding behavior (e.g., mate defence, copulation attempts). This may have resulted in koalas being active more frequently and for longer periods. 

 In our study, overall activity (ODBA) was higher during the early- rather than late-breeding season, which is consistent with breeding behaviour in this species. During the early-breeding season, most copulations occur and young are weaned; while in the late-breeding season, there are fewer copulations because most females are either pregnant or have recently given birth. In the early-breeding season, koalas moved farther and were active for slightly longer each day (see Table 3). Furthermore, anecdotal observations suggest that aggressive interactions between males, and copulation attempts were more frequent during this period. The relationship between ODBA and metabolic rate is yet to be validated for koalas; however, studies with other species suggest that ODBA is a reliable proxy for energy expenditure [20,21,23]. It is therefore likely that energy demands for koalas peak during the early-breeding season. Our study also suggests that male and female koalas partition activity differently with males investing more in mate selection and defence. Although the total amount of time spent in activity and ODBA did not vary with gender in either sampling period, males moved farther and changed trees more frequently than females.

 The duration of activity bouts was highly variable. The median length of bouts was relatively short (17.5 minutes) and did not vary with gender or season. However, some bouts were as long as 6.4 hours. Previous studies suggest that the majority of koala activity is associated with feeding [19,39,41], but in our study, longer bouts may also have involved movements between trees. Koalas generally moved between trees daily, although this was more frequently observed for males (see Table 3).

We observed a clear pattern of diel activity during both sample periods. Koalas were least active (less than 10 minutes per hour active) from sunrise (06:00 h) to mid-afternoon (14:00 h), after which activity increased to peak between 21:00 - 22:00 h (mean of 25 minutes per hour active; See Figure 3). Koalas are generally considered to be nocturnal, although daytime activity has been reported [11,19,39,41]. It has been suggested that the high levels of daytime activity observed in some studies may be due to study animals having advanced tooth wear causing them to feed for longer periods and to space feeding bouts throughout a 24-h period [19]. In a study where koalas were monitored with acoustic devices, nocturnal activity was reported in koalas with low-to-medium tooth wear, but activity throughout a 24-h period in koalas with advanced tooth wear [19]. Although we did not measure tooth wear as part of this study, other studies of the same population suggest that individuals with advanced tooth wear represent only a small proportion (<5%) of individuals (D. Whisson, unpublished data) such that tooth wear was unlikely to have had any influence on activity in our study.

 In both sampling periods, activity was correlated with hourly temperature, with activity increasing with declining ambient temperature. This is consistent with the species’ thermoregulatory properties [42]. Although koalas can endure cold conditions as a result of the insulative properties of their pelts [42], they are less able to tolerate hot conditions [5]. To minimise thermoregulatory stress, they therefore must limit their activity to cooler periods of the day [19].

 The chemical properties of koala food trees may also have influenced activity patterns. *Eucalyptus* foliage is high in formylated phloroglucinol compounds (FPCs), a group of plant secondary metabolites that inhibit total food intake [8]. Feeding bouts are interspersed with periods of inactivity to allow ingested material to be detoxified, and decline in duration after the first initial bout for the day [43]. The decline in activity after 22:00 h in our study may, therefore, be due to koalas needing to rest to digest food after the first main feeding bout of the afternoon or evening. 

 Our study has demonstrated the effectiveness of accelerometry for examining activity patterns of the koala. Because of the koala’s highly specialised and energy-poor diet, activity patterns of this species are likely to vary with changes in resource availability, climate variables, and seasonal energy requirements [12]. Accelerometry will therefore be a useful tool for measuring the impacts of anthropogenic- and climate-mediated environmental change on this species. Accelerometry also has potential application to other species and their behavioral adaptation to spatially and temporally varying environments. 
